# New Insights into the Impact of Human Papillomavirus on Oral Cancer in Young Patients: Proteomic Approach Reveals a Novel Role for S100A8

**DOI:** 10.3390/cells12091323

**Published:** 2023-05-05

**Authors:** Marisol Miranda-Galvis, Carolina Carneiro Soares, Carolina Moretto Carnielli, Jaqueline Ramalho Buttura, Raisa Sales de Sá, Estela Kaminagakura, Fabio Albuquerque Marchi, Adriana Franco Paes Leme, Clóvis A. Lópes Pinto, Alan Roger Santos-Silva, Rogerio Moraes Castilho, Luiz Paulo Kowalski, Cristiane Helena Squarize

**Affiliations:** 1 Laboratory of Epithelial Biology, Department of Periodontics and Oral Medicine, University of Michigan School of Dentistry, Ann Arbor, MI 48109, USA; mgalvis@augusta.edu (M.M.-G.); rcastilh@umich.edu (R.M.C.); 2 Oral Diagnosis Department, Piracicaba Dental School, University of Campinas (UNICAMP), Piracicaba 13414-903, SP, Brazil; carol_ccsm@hotmail.com (C.C.S.); raisadesa@hotmail.com (R.S.d.S.); alanroge@unicamp.br (A.R.S.-S.); 3 Department of Microbiology, Immune Biology, and Genetics, Center for Molecular Biology, University of Vienna, 1030 Vienna, Austria; 4 Brazilian Biosciences National Laboratory (LNBio), Brazilian Center for Research in Energy and Materials (CNPEM), Campinas 13083-970, SP, Braziladriana.paesleme@lnbio.cnpem.br (A.F.P.L.); 5 Laboratory of Bioinformatics and Computational Biology, A.C.Camargo Cancer Center (CIPE), São Paulo 01508-010, SP, Brazil; jaquelineramalho0@gmail.com; 6 Department of Bioscience and Oral Diagnosis, Science and Technology Institute, University of São Paulo State (UNESP), São José dos Campos 01049-010, SP, Brazil; estela.tango@unesp.br; 7 Center for Translational Research in Oncology, Cancer Institute of the State of São Paulo (ICESP), São Paulo 01246-000, SP, Brazil; 8 Comprehensive Center for Precision Oncology, University of São Paulo, São Paulo 05508-900, SP, Brazil; 9 Department of Anatomic Pathology, A.C.Camargo Cancer Center, São Paulo 01509-001, SP, Brazil; coipinto@uol.com.br; 10 Rogel Cancer Center, University of Michigan, Ann Arbor, MI 48109, USA; 11 Head and Neck Surgery Department, Medical School, University of São Paulo, São Paulo 05508-900, SP, Brazil; lp_kowalski@uol.com.br; 12 Department of Head and Neck Surgery and Otorhinolaryngology, A.C.Camargo Cancer Center, São Paulo 01509-001, SP, Brazil

**Keywords:** oral cancer, HPV, proteomics, prognosis, S100A8

## Abstract

Human papillomavirus (HPV) infection has recently been linked to a subset of cancers affecting the oral cavity. However, the molecular mechanisms underlying HPV-driven oral squamous cell carcinoma (OSCC) onset and progression are poorly understood. Methods: We performed MS-based proteomics profiling based on HPV status in OSCC in young patients, following biological characterization and cell assays to explore the proteome functional landscape. Results: Thirty-nine proteins are differentially abundant between HPV (+) and HPV (−) OSCC. Among them, COPS3, DYHC1, and S100A8 are unfavorable for tumor recurrence and survival, in contrast to A2M and Serpine1, low levels of which show an association with better DFS. Remarkably, S100A8 is considered an independent prognostic factor for lower survival rates, and at high levels, it alters tumor-associated immune profiling, showing a lower proportion of M1 macrophages and dendritic cells. HPV (+) OSCC also displayed the pathogen-associated patterns receptor that, when activated, triggered the S100A8 and NFκB inflammatory responses. Conclusion: HPV (+) OSCC has a peculiar microenvironment pattern distinctive from HPV (−), involving the expression of pathogen-associated pattern receptors, S100A8 overexpression, and NFκB activation and responses, which has important consequences in prognosis and may guide therapeutic decisions.

## 1. Introduction

The human papillomavirus (HPV) is a small, non-enveloped DNA virus that presents a tropism for basal keratinocytes of the stratified squamous epithelium, often detected in mucosal and cutaneous tissues [1,2,3,4,5]. More than 200 types of HPV have been identified and classified into five major genera: ∝-HPV, β-HPV, γ-HPV, mu-HPV, and nu-HPV. The ∝-HPV genus is categorized as low- and high-risk for developing malignant lesions [6,7,8]. High-risk HPV infection is mainly linked to cervical cancer, being responsible for 95% of cases. Nevertheless, HPV infection has also been linked to an alarming increase in HPV-positive head and neck cancers over time, currently surpassing the number of cases in cervical cancer [9,10].

High-risk HPV, particularly HPV 16 and 18, is mainly transmitted with sexual contact and is frequently found on the genital and oral mucosa [8,11,12]. The HPV oncoproteins E6 and E7 affect multiple cell functions and molecular signaling, including the inactivation and degradation of the tumor suppressor protein p53 and retinoblastoma protein (pRb), respectively [9,13,14,15]. Thus, the transcription of E6 and E7 prompts tumor formation via the suppression of cell cycle checkpoints and assists the replication of damaged DNA and the transformation and immortalization of epithelial cells [16,17,18,19]. Although E6 and E7 induce cells’ malignant transformation, additional genetic events are necessary to promote carcinogenesis and tumor progression, including those prompting HPV infection persistence and tumor immune evasion [20]. 

High-risk HPV has been linked to a subset of head and neck cancers affecting the oropharynx and oral cavity [21,22,23,24,25,26,27], which represents a distinct spectrum of the disease at clinical and molecular levels. The HPV-associated carcinomas in the head and neck region include a non-keratinizing squamous cell carcinoma with rising incidence in young men, who display higher survival rates than those with HPV-negative tumors [28,29,30,31]. HPV-associated tumors represent 70 to 90% of oropharyngeal carcinomas (OPC) [25,32,33]; in contrast, these tumors make up to 30% of the carcinomas found in the oral cavity, also known as oral squamous cell carcinoma (OSCC) [25,26,27]. Notably, the prevalence of HPV-associated OSCC is even higher in patients younger than forty years old, mainly when these tumors are positive for HPV16 [34]. Although little is known about head and neck cancer in younger patients, its incidence is gradually increasing [35,36].

While the role of the HPV infection is essentially known in OPC, the effects of HPV on oral cancer onset and outcomes remain poorly understood, especially in younger adults (e.g., 40 years and younger). A possible explanation for these contradictory results may be the consequence of heterogenous samples that assess oral cavity tumors, mainly cases located in the tongue, simultaneously with oropharynx samples. In addition, various studies evaluating the prevalence of HPV infection lack insights to determine the biological processes related to HPV (+) OSCC’s clinical behavior, even when the prevalence is low. Many pieces of evidence indicate that younger patients with cancer in the oral cavity may represent a distinct cohort and behavior of the disease. Here, we used mass spectrometry-based proteomics combined with bioinformatics and in vitro assays to identify, characterize, and analyze the proteome profile of HPV-associated tumors in specific locations of the oral cavity in young patients, who were uniform in terms of age, clinical stage, and survival rates. We determined a set of proteins that are important to HPV-associated OSCC. The proteomic-based identification was correlated with clinical significance and prognosis. Among the five proteins impacting patient survival, the role of S100A8 was highlighted after validation in a large cohort and linked to tumor-associated immune profiling.

## 2. Materials and Methods

### 2.1. Study Population

This retrospective study selected the OSCC cases positive for HPV among a cohort of patients who were aged up to 40 years old and treated at the A.C.Camargo Cancer Center (São Paulo, SP, Brazil) between 1970 and 2006. Data on sociodemographic characteristics (e.g., gender, age, smoking habits), clinicopathological features (i.e., TNM), histopathological grade, HPV status (i.e., HPV type and positivity), and survival rates (i.e., disease-free survival, cancer-specific survival, overall survival) were obtained. HPV status was previously determined using primers GP5+/GP6+ specific for the L1 gene of 17 HPV types using radiative probes and PCR assay for HPV16 [34]. Among 144 tumor samples analyzed, 15 OSCC cases were HPV16-positive in patients below 40 years old. A certified pathologist reviewed the samples and confirmed the diagnosis. Inclusion criteria included surgical samples before adjuvant treatment and no presence of a second primary tumor. Tumors affecting the lips and oropharynx were excluded. After applying the selection criteria, we established two experimental groups with nine HPV (+) OSCCs and 11 HPV (−) OSCCs. 

### 2.2. Laser-Capture Microdissection and Protein Extraction

Histological sections with 10 μm thickness from paraffin-embedded surgical specimens were placed on an Arcturus PENmembrane glass slide (Applied Biosystems^®^, Waltham, MA, USA), deparaffinized with xylene, and stained with hematoxylin (Sigma-Aldrich, Waltham, MO, USA). Neoplastic epithelial cells were microdissected using LMD CTR 6500 equipment (Leica Microsystems, Wetzlar, Germany). A mean area of ~4000 μm^2^ per sample was obtained. Proteins were extracted and digested as previously described [37]. In brief, samples were treated with 1.6 M urea, reduced with 5 mM dithiothreitol for 25 min at 56 °C, and alkylated with 14 mM iodoacetamide for 30 min at room temperature, protected from light. The proteins were digested with trypsin at 37 °C for 16 h. After adding 0.4% formic acid, the samples were dried in a vacuum concentrator and stored at −80 °C.

### 2.3. Liquid Chromatography Coupled with Tandem Mass Spectrometry (LC-MS/MS)

An aliquot containing 4.5µL of peptide mixture was analyzed on an LTQ Orbitrap Velos (Thermo Fisher Scientific, Waltham, MA, USA) mass spectrometer coupled to nanoflow liquid chromatography on an EASY-nLC system (Proxeon Biosystems, Odense, Dinamarca) through a Proxeon nanoelectrospray ion source. Peptides in 0.1% formic acid were separated using a 2–90% acetonitrile gradient in a PicoFrit analytical column (20 cm × ID75, 5 µm particle size, New Objective) with a flow rate of 300 nL/min over 212 min and a gradient of 35% acetonitrile at 175 min. The nanoelectrospray voltage was set to 2.2 kV and source temperature to 275°C. Instrument methods for the LTQ Orbitrap Velos were set up in the data-dependent acquisition mode. Full scan MS spectra (m/z 300–1600) were acquired in the Orbitrap analyzer after accumulation to a target value of 1e6. The resolution in the Orbitrap was set to r = 60,000, and the 20 most intense peptide ions with charge states ≥ 2 were sequentially isolated to a target value of 5000 and fragmented in the high-pressure linear ion trap through CID (collision-induced dissociation) with a normalized collision energy of 35%. Dynamic exclusion was enabled with an exclusion size list of 500 peptides, exclusion duration of 60 s duration, and repetition count of 1. An activation Q of 0.25 and activation time of 10 ms were used.

### 2.4. LC-MS/MS Data Analysis

The raw files were processed using the MaxQuant v1.3.0.3 software [38], and MS/MS spectra were searched against the Human UniProt database (release on 7 January 2015—89,649 sequences and 35,609,686 residues) using the Andromeda search engine [39]. A tolerance of 20 ppm was considered for precursor ions (MS search) and 0.5 Da for fragment ions (MS/MS search), with a maximum of 2 missed cleavages. The oxidation of methionine and protein N-terminal acetylation were set as variable modifications and carbamidomethylation of cysteine as fixed modification. Label-free quantification (LFQ) was used for protein quantification with a 2 min window for matching between runs and minimal ratio count set as 1. A maximum of 1% peptide and 1% protein FDR was considered. Statistical analysis was performed with Perseus v1.2.7.4 software [38], available with the MaxQuant package. Protein datasets were processed excluding reverse sequences and only identified by site entries. A filter of minimum valid values (3 valid values) was applied in at least one group, and Student’s *t*-test was used to assess significance to identify differentially expressed proteins (*p* < 0.05). 

### 2.5. Bioinformatics Analysis

Proteins with differential expression between the HPV− and HPV+ groups were submitted to an enrichment analysis to gain biological information from this list of the identified proteins. Using the Integrated Interactome System (IIS) platform [40], the UniProt IDs of differential proteins were submitted to perform enrichment analysis for the GO (Gene Ontology) [41] database. Only significant biological processes (*p*-value < 0.05) were considered in the results. Enrichment analysis was performed using the Enrichr tool [42,43], and canonical pathways were obtained using Ingenuity Pathway Analysis software (IPA; v8.0; Ingenuity^®^ Systems, Redwood City, CA, USA; http://www.ingenuity.com, accessed on 3 July 2017). MiRNA target prediction was performed using the miRWalk tool (http://mirwalk.umm.uni-heidelberg.de, accessed on 3 July 2017). Three databases were considered to identify the potential targets: Targetscan, miRDB, and miRTarBase. A binding score value higher than 0.95 was defined as the cut-off. The immune cells infiltration profile was estimated through a deconvolution method based on gene expression panels to determine the proportion of B cells, CD4+ T cells, CD8+ T cells, natural killer (NK) cells, macrophages, dendritic cells (DC), and neutrophils in the samples from The Cancer Genome Atlas (TCGA) HNSCC, selecting oral cavity samples (N = 301). The reference signature matrix was constructed using the TIMER pipeline [44] and LM22 marker genes proposed by Newman et al. [45]. The deconvolution algorithm CIBERSORT [45] was then used to estimate relative proportions of immune cells above 1000 permutations and disabled quantile normalization as set parameters. The proportion of the immune profile was compared between “top” and “bottom” samples and classified by the third and first quartile, according to S100A8 expression. The Wilcoxon test statistical significance was set at *p*-value < 0.05. A second approach, known as xCell [46], was based on gene set enrichment analysis and used to reinforce the findings of immune cell comparison between “top” and “bottom” samples. Genomic data analysis to identify TRL mutations and HPV status was extracted from publicly available data [47] using the cBioPortal for Cancer Genomics (v4.1.2) [48,49].

### 2.6. Cell Lines and TLR Activation

The cell lines derived from human tongue squamous cell carcinoma and positive for HPV16, UPCI-SCC154, and UM-SCC104 (gift from Drs. S.M. Gollin and T.E. Carey) were grown in minimum essential medium and 10% FBS. The medium was supplemented with 50 mg/mL gentamicin (Sigma-Aldrich, St. Louis, MO, USA), 200 mM L-glutamine, and MEM non-essential amino acid solution (ThermoFisher Scientific, Waltham, MA, USA). The HPV16-negative cell lines derived from human tongue squamous cell carcinoma, WSU-HN6 and WSU-HN13, were cultured in Dulbecco’s modified Eagle’s medium (DMEM; Sigma-Aldrich, St. Louis, MO, USA) with 10% FBS and 1% antibiotic/antimycotic solution (Sigma-Aldrich, St. Louis, MO, USA). Cell lines were maintained at 37 °C in a humidified incubator with 5% CO_2_. Where indicated, cells were grown to a maximum of 40% confluence, starved for 24 h, and treated with 10 ng/mL of synthetic monophosphoryl lipid A (MPLA) for 24 h. 

### 2.7. Immunofluorescence

Immunofluorescence staining was performed on cells seeded in glass slides following 4% PFA fixation. Tissue sections from a tissue microarray (TMA) containing 91 OSCC (cohort 2) were also stained, as previously described [50]. The standard protocol was performed to deparaffinize and rehydrate the tissues through graded ethanol solutions, followed by antigen retrieval with 10 mM citric acid then 1% BSA/PBS blocking. Primary antibodies (Anti-MRP8 antibody (Abcam Plc, Cambridge, UK) or Rel-A (PCRP-RELA-2B6, Developmental Studies Hybridoma Bank, IA, USA)) were incubated overnight at 4 °C. Slides were washed with PBS and incubated with a secondary antibody, Alexa Fluor 568 or Alexa Fluor 488 (ThermoFisher Scientific, Waltham, MA, USA), and Hoechst 33342. Images were visualized using a Nikon Eclipse 80i microscope (Nikon, Melville, NY, USA) and analyzed with the NIS Element software. Cell counting was performed on ten fields of each slide. The results are shown as number of positive cells by the total number of cells identified with nuclei counting. They were classified into the following groups: 0 (no positive cells), 1 (1–10% positive cells), 2 (10–50% positive cells), and 3 (>50% positive cells). Additionally, the cases were divided into low (≤50% positive cells) and high expression (>50% positive cells) for statistical purposes.

### 2.8. Flow Cytometry

After trypsinization, cell suspensions were adjusted to a concentration of 1 × 10^6^ cells/mL and fixed with cold 1% PFA. The cells were incubated with the anti-TLR4 antibody (76B357.1, Abcam Plc, Cambridge, UK) for 45 min in RT using a moving rotor in addition to Alexa Fluor-488. The cells were analyzed using a BD Accuri C6 Plus flow cytometer (BD Biosciences, Franklin Lakes, NJ, USA).

### 2.9. Statistical Analysis

Statistical analyses of associations between variables were performed using Fisher’s exact test, and for continuous variables, the non-parametric Mann–Whitney U test was used. The Kaplan–Meier method was used to analyze survival probabilities, while multivariate analysis was performed using the Cox proportional hazards model. The log-rank test was applied to assess the significance of differences among actuarial survival curves with a 95% confidence interval. A significance set to *p* < 0.05 was adopted. Analyses were performed using the IBM Software SPSS statistics (V.23.0—Armonk, NY: IBM Corp.) and GraphPad Prism (V.5.0—San Diego, CA, USA).

## 3. Results

### 3.1. Determination of Differently Expressed Proteins between HPV (+) and HPV (−) OSCC through Discovery of Proteomics

**Figure 1:**
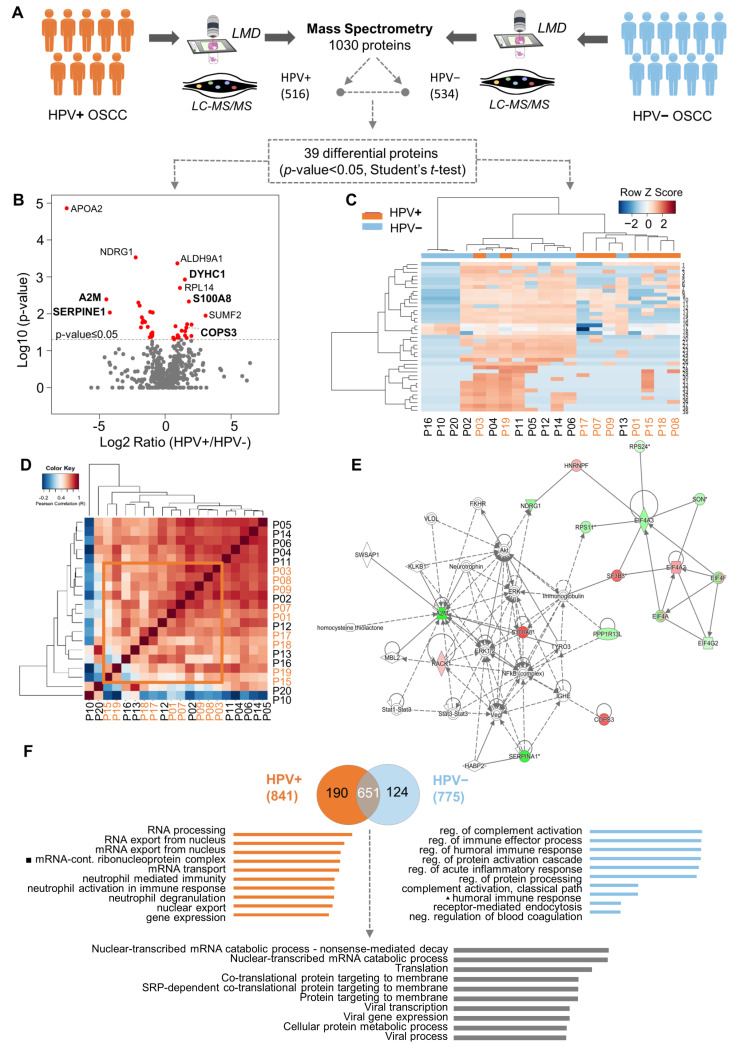
Quantitative proteome profiling revealed thirty-nine proteins differentially expressed between HPV− and HPV+ oral squamous cell carcinoma (OSCC) and enriched for biological processes. (**A**) Proteomics analysis was performed in cohort 1, composed of eleven HPV− (blue) and nine HPV+ (orange) OSCC-affected patients younger than forty years old at an advanced clinical stage. Laser-capture microdissection was carried out in the paraffin-embedded surgical specimens to isolate the neoplastic cells. The Venn diagram shows the common and “exclusive” proteins identified. (**B**) Intensity ratios (log2 ratio of the LFQ intensity, HPV+/HPV−) of proteins identified in patients’ samples represented in volcano plots. Red dots indicate differentially abundant proteins (*p*-value < 0.05, Student’s *t*-test, two-sided). (**C**) Clustering analysis of all identified proteins in HPV− and HPV+ samples. Proteins (rows) and tumor samples from patients (denoted as “P”, columns) are colored based on the protein abundance (Z-scored log2 LFQ intensity values). HPV− or HPV+ tumor samples are indicated in blue or orange, respectively. Hierarchical clustering was performed using Canberra distance with the Ward method. (**D**) Pearson correlation coefficients derived from pairwise comparison of HPV− and HPV+ tumor samples from patients (P01-P20). Log2 LFQ intensity values of the protein dataset after filtering reverse and ‘only by site’ entries were used to calculate the correlation coefficient using Perseus software. The dendrogram was built using Euclidean distance with complete ligation. (**E**) Protein interactions for differentially expressed proteins revealed a network focused on NFκB. Upregulated (red) and downregulated (green) proteins in HPV+ tumors were correlated with other molecules (white) using Ingenuity Pathway Analysis. (**F**) The ten most-enriched biological processes (Gene Ontology) for “exclusive” proteins from the HPV− and HPV+ samples and for abundant differential proteins between the two conditions (two-sided Student’s *t*-test, adjusted *p*-value < 0.05) are represented. Abbreviations: LMD, laser microdissection; LC-MS/MS, liquid chromatography–tandem mass spectrometry.

To identify which proteins are associated with the HPV (+) OSCC, we compared the proteome profiles of nine HPV (+) OSCCs against 11 HPV (−) OSCCs using quantitative mass spectrometry (Figure 1A). Samples selected for the proteomics analysis had no significant differences between the two defined patient groups regarding the sociodemographic and clinicopathological features, clinical stage, age, and survival rates (Supplementary Table S1 and Supplementary Figure S1). As a result, we decreased the noise and bias among the groups, which allowed us to determine the differences caused by HPV tumorigenesis. We mapped the proteome of the groups, which yielded the identification of 1030 proteins. After excluding the reverse peptide sequences, entries that were identified as ‘only by site’ were also excluded. Therefore, 987 proteins were identified, of which 535 were confidently quantified samples (filter of three in at least three valid values in one or more groups). Of these, 518 were present in both groups, 1 was exclusive to HPV (+) tumors, and 16 were exclusive to the HPV (−) tumors. Thirty-nine proteins were selected as differentially abundant between the OSCC HPV (+) and HPV (–) groups (*p*-value ≤ 0.05, Student’s *t*-test) (Figure 1A,B). The proteins highly expressed in the HPV (+) cohort were RACK1 (receptor of activated protein C kinase 1), CAD (CAD protein), RPL14 (60S ribosomal protein L14), RPL29 (60S ribosomal protein L29), EIF4A2 (eukaryotic initiation factor 4A-II), HNRNPF (heterogeneous nuclear ribonucleoprotein), SF3B3 (splicing factor 3B subunit 3), VARS (valine-tRNA ligase), DYHC1 (cytoplasmic dynein 1 heavy chain 1), LRPPRC (leucine-rich PPR motif-containing pro), CKAP4 (cytoskeleton-associated protein 4), COPS3 (COP9 signalosome complex subunit 3), and S100A8 (protein S100-A8). On the other hand, RPL23 (60S ribosomal protein L23), RPS11 (40S ribosomal protein S11), RPS24 (40S ribosomal protein S24), EIF4A3 (eukaryotic initiation factor 4A-III), NDRG1 (protein NDRG1), EIF4G2 (eukaryotic translation initiation factor, PLP2 (proteolipid protein 2), SERPINA1 (alpha-1-antitrypsin), A2M (alpha-2-macroglobulin), HP1BP3 (heterochromatin protein 1-binding prot), SON (protein SON), PPP1R13L (RelA-associated inhibitor), and YWHAH (14-3-3 protein eta) were favored in the HPV (−) cohort (Figure 1B). Note that the hierarchical clustering and Pearson correlation coefficients derived from the pairwise comparison allowed the visualization of protein quantification across all HPV (−) and HPV (+) specimens (Figure 1C,D) (Supplementary Figure S2). Clustering analysis revealed that most samples were grouped according to HPV status, indicating a particular proteomic profile. Pearson correlation was uniform, varying only between −0.2 and 1. The compressive canonical pathway analysis, using the bioinformatics tool Ingenuity^®^ Pathway Analysis (IPA^®^) software program v01.12, indicated that the NFκB pathway and AKT are relevant in the proteome (Figure 1E and Supplementary Figure S1D). Moreover, the enrichment analysis in the Gene Ontology database indicated 26 proteins with significant biological processes (Supplementary Table S2, * *p*-value ≤ 0.05). The ten most-enriched biological processes were determined using mutually exclusive proteins, and differentially abundant proteins were found in both conditions (Figure 1F). These proteins were characterized for biological processes related to virality, such as viral infection, transcription, and translation (adjusted *p*-value < 0.05). GO terms for RNA processes were overrepresented for proteins that were identified and differentially abundant in HPV (+) tumors. In contrast, the immune process regulation was augmented in HPV (−) tumors (Figure 1F and Supplementary Table S3).

Furthermore, to explore the role of the identified proteins in oral carcinogenesis and tumor progression, we performed an integrative analysis of miRNAs as possible regulatory mechanisms of the identified proteins using samples from the TCGA. We compared the miRNA profile of the targeted proteins in the oral cavity tumors with adjacent non-neoplastic tissue. The data showed 12 proteins presenting a fold change greater than 1.5: RPL14, EIF4A2, LRPPRC, CKAP4, COPS3, S100A8, NDRG1, EIF4G2, PLP2, Serpina1, A2M, and HP1BP3 (Supplementary Table S2).

### 3.2. High Expression of S100A8 Is Correlated with Advanced Clinical Stage and Worse Survival Rate

**Figure 2:**
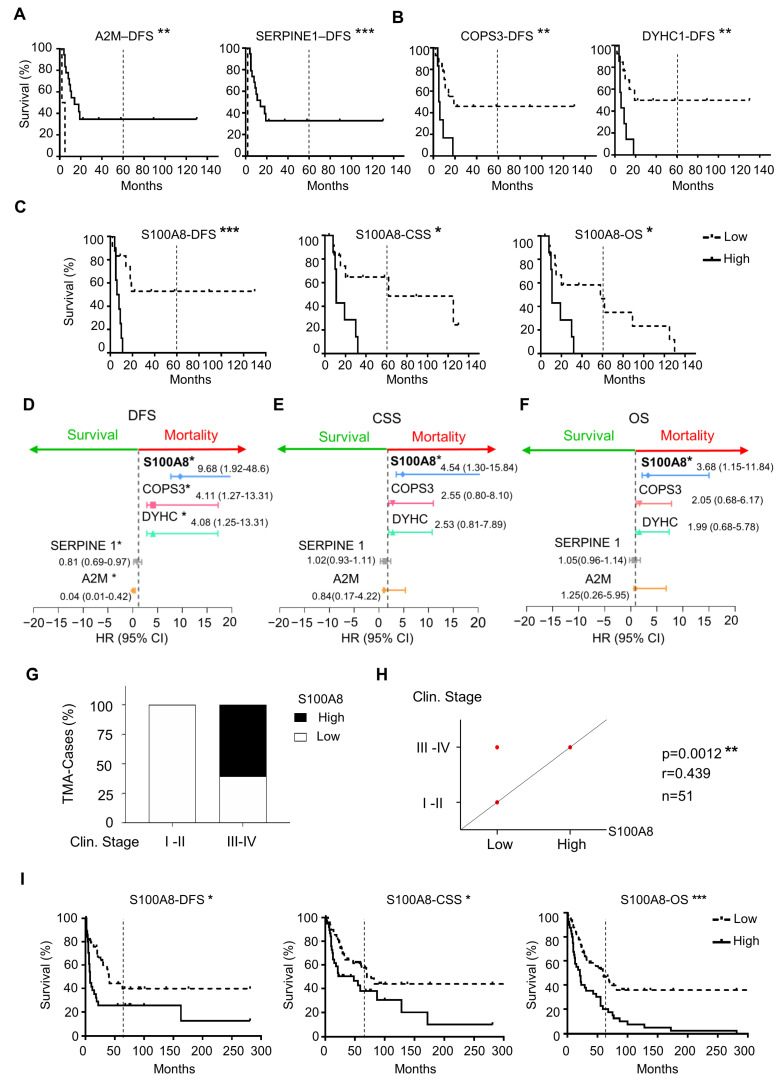
Correlation analysis of differentially expressed proteins with prognosis showed five proteins associated with survival outcomes. (**A**) The results from proteomics analysis were correlated with disease-free survival (DFS), cancer-specific survival (CSS), and overall survival (OS). Expression of A2M (** *p* = 0.0015) and Serpine1 (*** *p* < 0.0001) was associated with an increase in DFS (**B**), while COPS3 (** *p* = 0.006), DYHC1 (** *p* = 0.006), and S100A8 (*** *p* = 0.0005) were associated with decreasing DFS. (**C**) S100A8 was the only protein that influenced CSS (* *p* = 0.0109) and OS (* *p* = 0.0215). (**D**) Cox proportional regression analysis indicated A2M (** *p* = 0.01), Serpine1 (** *p* = 0.01), COPS3 (** *p* = 0.01), DYHC1 (** *p* = 0.01), and S100A8 (** *p* = 0.01) to be significant independent predictors for DFS. (**E**) S100A8 was significant for CSS (* *p* = 0.017) and (**F**) OS (* *p* = 0.02). (**G**) Cohort 2 with ninety-one OSCCs was used to corroborate the proteomics results. (**H**) High levels of S100A8 were correlated with advanced clinical stage (*** *p* = 0.0012, n = 51) (**I**) and decreased DFS (** *p* = 0.005), CSS (** *p* = 0.0109), and OS (* *p* = 0.0215). Asterisk denotes significant differences (ns *p* > 0.05, * *p* ≤ 0.05, ** *p* < 0.01, *** *p* < 0.001).

To explore the clinical relevance of the differently expressed proteins between HPV (+) and HPV (−), we used Kaplan–Meier survival analysis to identify the proteins with prognostic value within our patient cohort. Notably, of 26 differential expressed proteins, five were associated with survival outcomes that were independent of HPV status. High expression of A2M and Serpine1 was correlated with an increase in the DFS (** *p* = 0.0015, *** *p* < 0.0001, respectively) (Figure 2A). On the other hand, high expression of COPS3 (** *p* = 0.006) and DYHC1 (** *p* = 0.006) was associated with decreased DFS (Figure 2B). Notably, S100A8 overexpression was the protein associated with all three assessments, including the decrease in CSS (* *p* = 0.0109), DFS (*** *p* = 0.0005), and OS (* *p* = 0.0215) (Figure 2C). Interestingly, the estimated OS rate for patients with low expression of S100A8 was 48% at five years, while patients with high expression of S100A8 did not survive the 5-year endpoint. The multivariate Cox proportional regression analysis estimated the hazard ratio (HR) and identified the independent prognostic factors with respect to the risk of recurrence and death for these identified markers. Our results indicated that A2M (** *p* = 0.01), Serpine1 (** *p* = 0.01), COPS3 (** *p* = 0.01), DYHC1 (** *p* = 0.01), and S100A8 ** (*p* = 0.01) were significant and independent predictors for DFS (Figure 2D). Once again, S100A8 was significant for CSS (* *p* = 0.017) and OS (* *p* = 0.02) (Figure 2E,F). Low expression of S100A8 showed a longer OS rate. Indeed, patients with high expression of S100A8 had a risk of death 3.68 higher than those with low expression. As we increased the sample size analysis using a second cohort of 91 OSCC, the prognosis impact of S100A8 was further confirmed. The results also indicated that higher levels of S100A8 were correlated with enlarged tumor size (* *p* = 0.030), advanced clinical stage (** *p* = 0.007), and positive surgical margins (* *p* = 0.015) (Figure 2G,H, and Supplementary Table S4). Patients with high expression of S100A8 showed lower DFS (* *p* = 0.020), CSS (* *p* = 0.023), and OS (*** *p* = 0.001) (Figure 2I). Aligned with our previous results, the high activity of S100A8 showed 30% OS in 5 years, compared with the 52% OS that resulted from the low activity of S100A8.

### 3.3. OSCCs with S100A8 Overexpression Exhibit Lower Amounts of M1 Macrophages and Dendritic Cells

**Figure 3:**
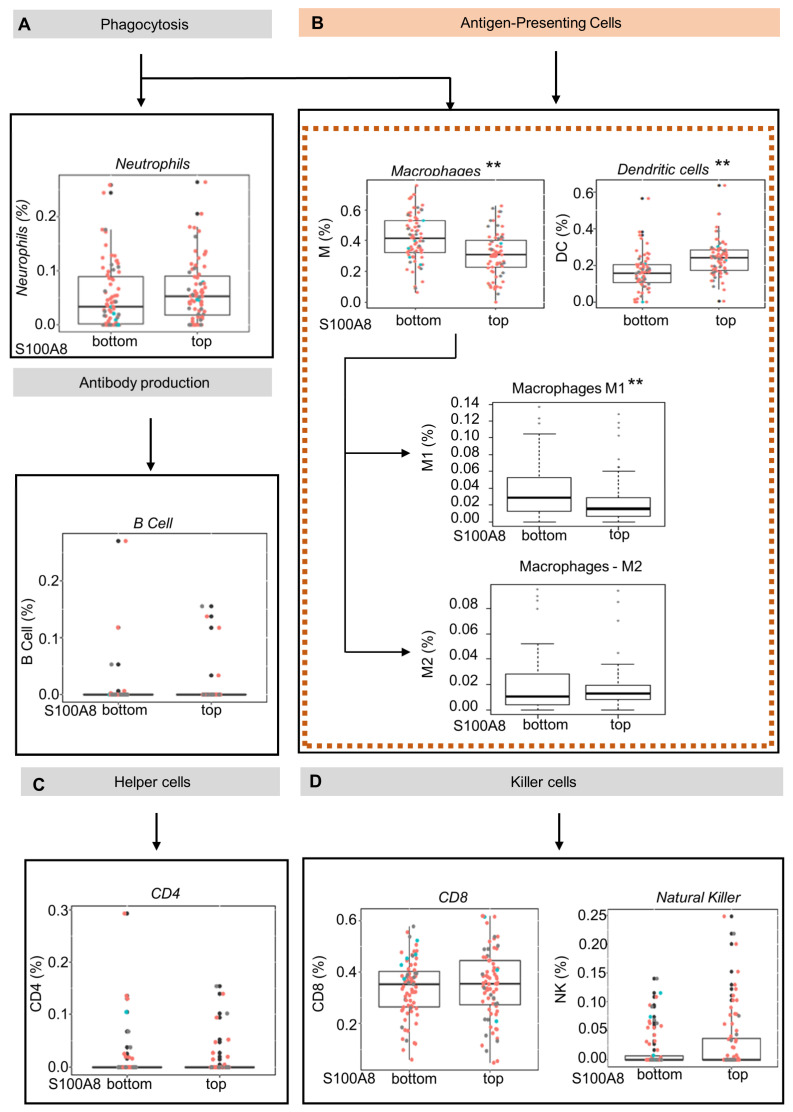
Oral squamous cell carcinoma with high levels of S100A8 mRNA exhibits a lower proportion of M1 macrophages and dendritic cells. (**A**–**D**) Using the CIBERSORT deconvolution algorithm and xCell enrichment analysis, we evaluated the tumor-infiltrating immune cells present in 152 OSCC (HPV+ (red), HPV−(blue), and unknown (black)) from the Cancer Genome Atlas database. The patients were sorted into top and bottom groups for S100A8 gene expression using quartiles of 0.75 (top) and 0.25 (bottom). (**B**) CIBERSORT analysis indicated that patients at the top for S100A8 had a lower proportion of antigen-presenting cells, macrophages (0.31% vs. 0.41%, ** *p* < 0.001), and immature dendritic cells (0.18% vs. 0.23%, ** *p* < 0.001). The xCell approach identified the specific subtype of cells differently contained: M1 macrophages (0.02% vs. 0.03%, ** *p* < 0.001) and dendritic cells ((**D**,**C**); 0.16% vs. 0.18%, *p* < 0.001).

S100A8 is a protein member of the S100 family, which regulates inflammatory processes and immune responses [51]. We next explored whether the upregulation of S100A8 influences the inflammatory cell infiltration in OSCC tumors by using the CIBERSORT gene expression-based deconvolution algorithm in the TCGA database. The patients were sorted in terms of up- or downregulation for S100A8 gene expression using quartile classification: top ≥ 75% (Q3) and bottom ≤25%(Q1). Considering the samples with RNA-Seq data, a total of 301 patients were identified. This classification generated groups with 75/76 patients, respectively. The analysis considered seven cell types: lymphocyte B, lymphocyte TCD4, lymphocyte TCD8, NK cell, neutrophil, macrophage, and dendritic cell (DC). The findings indicated that the abundance of antigen-presenting cells was inversely correlated with S100A8 expression (Figure 3A,D). Tumors from patients in the top quartile classification of S100A8 gene expression contained a significantly lower proportion of macrophages compared with the tumors from patients in the bottom quartile. (0.31% vs. 0.41%) (Figure 3B, ** *p* < 0.001). Similarly, the proportion of dendritic cells in the bottom quartile was 0.18%, while the top quartile contained 0.23% (Figure 3B, ** *p* < 0.001). Given the diversity function between cell subpopulations, we extended our analysis to identify the altered subtypes with an xCell as a secondary approach based on the gene set enrichment analysis. We determined that a lower proportion of M1 macrophages (0.02% vs. 0.03%, ** *p* < 0.001), which are known to activate innate immunity, was found in the tumors from patients in the top quartile classification of S100A8 gene expression, the same group that displayed higher DC cells (0.22% vs. 0.18%, ** *p* < 0.001). Additionally, we found a trend showing that the proportion of M2 macrophages, which are in charge of local immunosuppression, was higher in tumors from patients in the top quartile for S100A8 compared with the tumors from patients in the bottom quartile (0.01 vs. 0.018).

### 3.4. S100A8 Is Overexpressed in HPV-Associated OSCC

**Figure 4:**
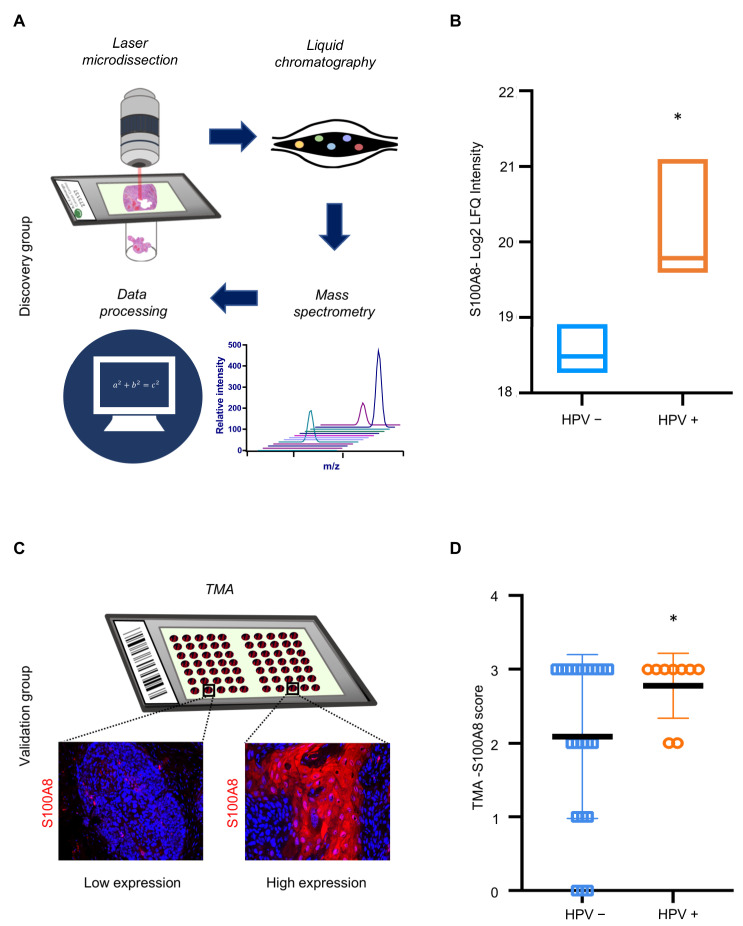
HPV+ OSCC exhibits high levels of S100A8 upon verification in an expanded cohort. (**A**) Samples were microdissected and submitted to proteomics and data analysis. Proteomics analysis in cohort 1 identified a significantly higher expression of S100A8 in HPV+ tumors than in tumors negative for HPV (* *p* = 0.004). (**B**) To further validate our findings regarding the S100A8 in HPV+ tumors, we used a larger cohort (cohort 2). Representative images of S100A8 in the cytoplasm of tumor cells and inflammatory cells in the stroma. (**C**) The cases were sorted into low (≤50% positive cells) and high (>50% positive cells) for S100A8 tumor-associated expression. (**D**) Quantitative analysis showed higher levels of S100A8 in HPV+ tumors (* *p* ≤ 0.05).

The results indicated that S100A8 is associated with immune infiltration and has an important prognostic significance for patients affected by OSCC. Notably, our proteomics analysis identified a significantly higher expression of S100A8 in HPV (+) tumors compared to HPV (−) tumors (* *p* ≤ 0.05) (Figure 4A,B and Supplementary Table S2). Next, we investigated whether S100A8 correlates with HPV tumor status. The data demonstrated the presence of S100A8 in tumor cells, especially in the cytoplasm, along with nuclear staining (Figure 4C). Aligned with the proteomics results, S100A8 was significantly overexpressed in the HPV (+) OSCC (n = 59, * *p* = 0.03) (Figure 4D). These results corroborate that HPV (+) OSCC presents higher levels of S100A8 protein.

### 3.5. HPV-Positive Cells Increase S100A8 and NFκB Activation upon Stimulation of Pattern Recognition Receptor TLR4

**Figure 5:**
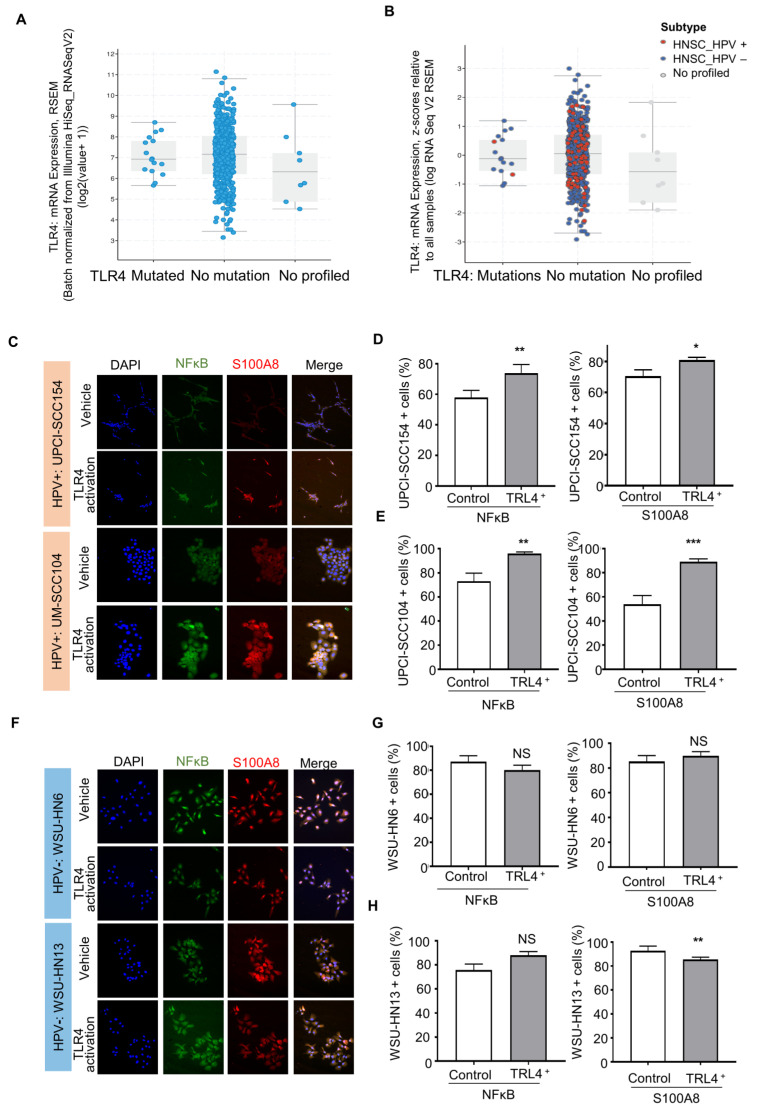
Activated TLR-4 upregulates NFκB and S100A8 in HPV+ oral squamous cell carcinoma (OSCC). (**A**). The graphs depict the number of cases and gene expression distributed among the mutation status of the pathogen recognition receptor TRL4. Most cases (96%) presented TRL4 in the non-mutated diploid occurrence, and further analysis included the HPV status (**B**). These analyses involved 515 cases from the TCGA database. (**C**–**E**) Furthermore, activation of TRL4 significantly increased the levels of NFκB and S100A8 in HPV+ OSCC cells, but not in HPV− OSCC cells. (**F**–**H**) Asterisk denotes significant differences (NS, not significant, * *p* < 0.05, ** *p* < 0.01, *** *p* < 0.001, two-sided Student’s *t*-test).

The IPA analysis revealed that S100A8 interacts with NFκB signaling. Aligned with our results, studies in breast cancer suggested that S100A8 is associated with tumor growth and invasion in breast cancer through the activation of the TLR4, RAGE, and NFκB pathways [52]. It is well known that Toll-like receptors (TLRs) are an important component in the immune response. Nevertheless, recent reports show that TLRs, particularly TLR4, are involved in the tumor initiation and progression of cancers, such as cervical cancer [53,54]. TLRs recognize pathogen-associated patterns triggered by viruses, bacteria, and other infections. The data from TCGA showed that TLR4 is expressed in head and neck cancer as a non-mutated diploid occurrence (~96%) (Figure 5A,B), indicating that the TRL receptor can serve as a functional receptor, especially in HPV (+) tumors. We further evaluated whether HNSCC cell lines express TRL4. The results showed expression of TRL4 in HPV (−) (WSU-HNSCC 6 and 13) and HPV (+) (UPCI-SCC154 and UM-SCC104) OSCC cell lines (Supplementary Figure S3), and the activation of TLR4 displayed different responses among cell lines based on the HPV status (Figure 5C–G). The HPV (+) cells significantly increased NFκB and S100A8 responses (Figure 5C–E), while S100A8 and NFκB were not dependent on TRL4 in HPV (−) cells (Figure 5F–H). 

**Figure 6:**
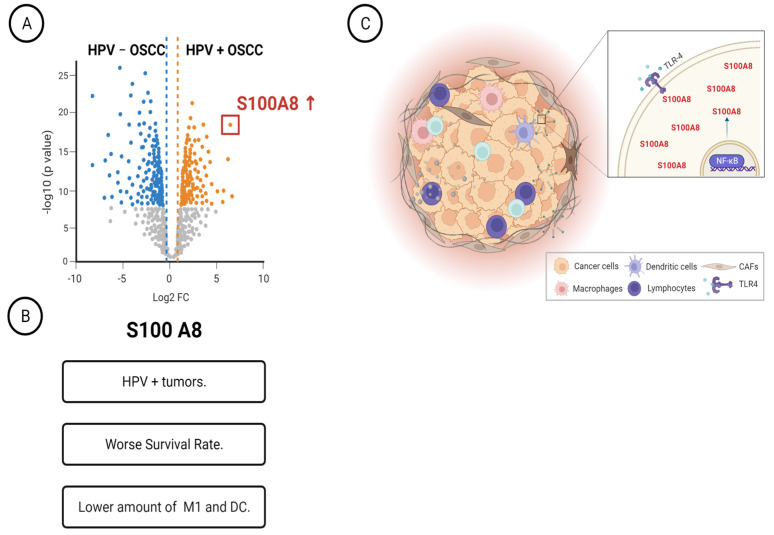
Schematic representation summarizing our main findings. (**A**) Characterization of HPV+ OSCC identified twenty-nine differentially expressed proteins. (**B**) The identified protein S100A8 was overexpressed in HPV-associated OSCC, and it was associated with worse survival rates and low counts of M1 macrophages. (**C**) HPV+ OSCC responds to TLR4 stimulation, increasing the levels of S100A8 and NF B.

Overall, our proteomics data combined with clinicopathological features indicated that the A2M, Serpine1, COPS3, DYHC1, and S100A8 proteins are associated with OSCC relapse, while S100A8 is also an independent prognostic factor for overall survival. The comparison of proteome profile based on HPV status revealed high S100A8 levels in HPV (+) tumors. Immune characterization determined that the bottom quartile of M1 macrophages and dendritic cells in tumors showed high levels of S100A8. Interestingly, HPV (+) OSCC tumor cells expressed the pathogen-associated pattern receptor, which, when activated, triggered S100A8 and NFκB activation and responses (Figure 6).

## 4. Discussion

OSCC has low survival rates worldwide, which is alarming because half of the newly diagnosed patients will die in five years, even after receiving multimodality approaches [55]. A better understanding of the agents involved in the establishment and progression of oral cancer holds promise in terms of creating preventive and novel personalized therapies tailored towards the oncogenic drivers and the modulation of the immune microenvironment. Thereby, it will be possible to identify patients who could benefit from a specific therapy, reducing the treatment failure rates and improving the survival rates [56]. In addition to the well-known risk factors of smoking and drinking, HPV infection was recently determined as an etiological factor in OSCC [57,58,59]. 

Nevertheless, little is known about the molecular circuitries present in HPV (+) OSCC. Using mass spectrometry-based proteomics, we characterized the proteome profile of OSCC and identified a panel of potential signature proteins in HPV (+) OSCC. We further evaluated the clinical significance of the 39 differentially expressed proteins and determined that five have prognostic value. While high levels of COPS3, DYHC1, and S100A8 were unfavorable for tumor recurrence, low amounts of A2M and Serpine 1 were associated with better DFS. Moreover, high levels of S100A8 are considered as an independent prognostic factor for lower survival rate, which correlates with the bottom quartile of M1 macrophages and dendritic cells in the tumor immune microenvironment. Furthermore, the HPV (+) OSCC tumor cells expressed the pathogen-associated pattern receptor, which, when activated, promotes S100A8 and NFκB inflammatory responses. 

The abnormal function of the NFκB protein is involved in tumorigenesis, stimulating cell proliferation, inhibiting apoptosis, and favoring angiogenesis and metastasis [60]. Most recently, S100A8 has emerged as an immunogenic protein linked to cancer [61,62]. It has been demonstrated that S100A8 is altered in cervical, lung, anaplastic thyroid, and breast cancers. S100A8 was correlated with cell proliferation, tumorigenesis, and metastasis, especially when it interacts with RAGE and other signaling proteins, such as p38, ERK1/2, JNK, and NFκB [52,63,64,65]. Recent work in cervical cancer has established that the HPV oncogenes E6/E7 increased the expression of a large panel of pro-inflammatory genes and S100A8, which ultimately condition the microenvironment to support tumor progression [66,67]. 

Other newly identified, survival-determining proteins have specific biological functions. The A2M protein has essential roles in inactivating a broad spectrum of proteases, transporting cytokines, and binding to growth factors and hormones [68]. Similar to plasma proteins, A2M is synthesized in the liver, while other cell types such as macrophages, fibroblasts, and adrenocortical cells carry out complementary local production in tissues [69]. Our interest in this study is based on the fact that A2M has an anti-inflammatory role in inhibiting pro-inflammatory cytokines and disrupting inflammatory cascades [68]. In addition, A2M presents anti-tumorigenic activity that acts as an inhibitor of central signaling pathways (e.g., phosphatidylinositol 3-kinase (PI3K), protein kinase B (AKT), and SMAD) and modulates tumor cell adhesion, migration, and growth [70]. Similar to A2M, SERPINA 1 is a serine protease inhibitor involved in regulating serine proteases and acts as an anti-inflammatory protein [71]. Despite this anti-inflammatory activity, the function of SERPINA 1 in tumor onset and progression is a matter of investigation due to many contradictory reports [72]. 

Two other proteins identified in our study were COPS3 and DYHC1; both were associated with decreased DFS. Previous studies showed that COPS3 activity regulates processes related to carcinogenesis and cancer progression, constitutively blocking photomorphogenic 1-mediated p53 degradation [73]. High levels of the COPS3 protein have been reported in different cancers, including oral and oropharyngeal carcinoma. Aligned with our study, it also correlated with survival outcomes [74]. COPS3 is a protein unit of the COP9 signalosome complex (CSN) with important functions in a broad spectrum of cellular processes, such as cell cycle control, signal transduction, and apoptosis [75]. Few studies have explored the proteome in head and neck tumors, including p16-positive tumors, and some used cell lines and oropharyngeal tumors [28,76,77]. Interestingly, we found similarities with five proteins identified in our study: SON, NDRG1, Serpin 1, RPL14, and 40S ribosomal protein S24. Furthermore, five ribosomal proteins (RPs) were highly expressed in our samples. RPL14 (60S ribosomal protein L14) and RPL29 (60S ribosomal protein L29) were expressed in HPV+ tumors, while (60S ribosomal protein L23), RPS11 (40S ribosomal protein S11), and RPS24 (40S ribosomal protein S24) were found in the HPV− group. Ribosomal proteins are essential in ribosome assembly, protein translation, and other ribosome-independent functions. Interestingly, alterations in RPs are involved in various pathological processes such as hematologic malignancies and solid tumors [78]. Studies also associated RP with the development and progression of breast and ovarian cancers, melanoma, multiple myeloma, leukemia, and glioblastoma, among others [79], suggesting that targeting ribosome biogenesis may lead to novel cancer treatment approaches [79]. Nevertheless, our results may serve as a base for further studies in this field.

It is important to note that our study has sample groups composed exclusively of human tumors derived from subsites within the oral cavity of young patients. This sample selection allows us to exclude the tumors found at the base of the tongue, a subsite anatomically contiguous to the oral cavity and oropharynx that has considerable differences regarding the sensibility for HPV infection in the oral cavity. We also compared the proteome profiles of tumors from patients with uniform ages, clinical stages, and survival rates, reducing noise and bias in the profile analysis. In addition, we combined powerful techniques, including mass spectrometry, bioinformatics, protein expression validation, and in vitro assays, to elucidate the underlying mechanisms involved in HPV (−)-related tumors and understand their clinical impact. 

In summary, we find that HPV (+) OSCC has a peculiar microenvironment pattern distinctive from HPV (−), involving the expression of pathogen-associated pattern receptors, S100A8 overexpression, and NFκB activation and responses, which has important consequences in prognosis and may guide therapeutic decisions.

## Data Availability

All proteomics LC-MS/MS raw data have been deposited in the ProteomeXchange Consortium via the PRIDE [80] partner repository with the dataset identifier PXD041856.

## Supplementary Materials

The following supporting information can be downloaded at: https://www.mdpi.com/article/10.3390/cells12091323/s1, Figure S1: Survival curves according to the HPV status and enlarged Ingenuity Pathway Analysis graph; Figure S2: Clustering analysis of all identified proteins in HPV+ and HPV− samples. Figure S3: TLR4 expression in cell lines. Table S1. Sociodemographic and clinicopathological features of the patients included in the proteomics analysis; Table S2: Differentially expressed proteins between HPV+ and HPV– OSCC samples after Gene Ontology enrichment analysis and miRNA prediction using samples in TCGA; Table S3: Enriched biological processes *p*-values; Table S4: Correlation between S100A8 expression and clinicopathological features.
