# Supplementary material for: New Insights into the Impact of Human Papillomavirus on Oral Cancer in Young Patients: Proteomic Approach Reveals a Novel Role for S100A8
Source: Cells. 2023 May 5;12(9):1323. doi: 10.3390/cells12091323 (PMC10177374; doi:10.3390/cells12091323)
Supplement: Supplementary file 1 [file cells-12-01323-s001.zip › cells-2301641-supplementary.pdf]

Supplementary Figure 1

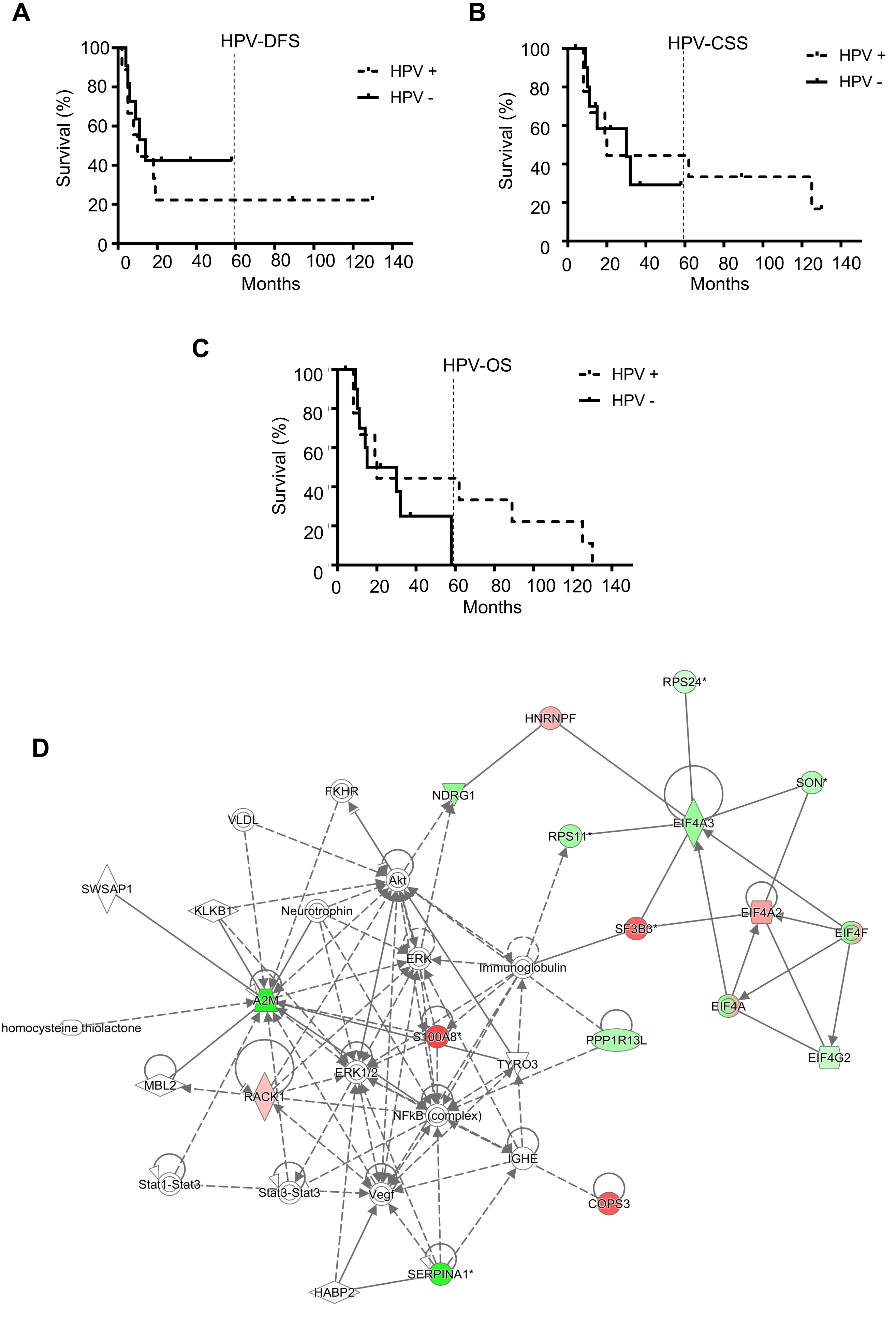

Supplementary Figure 2

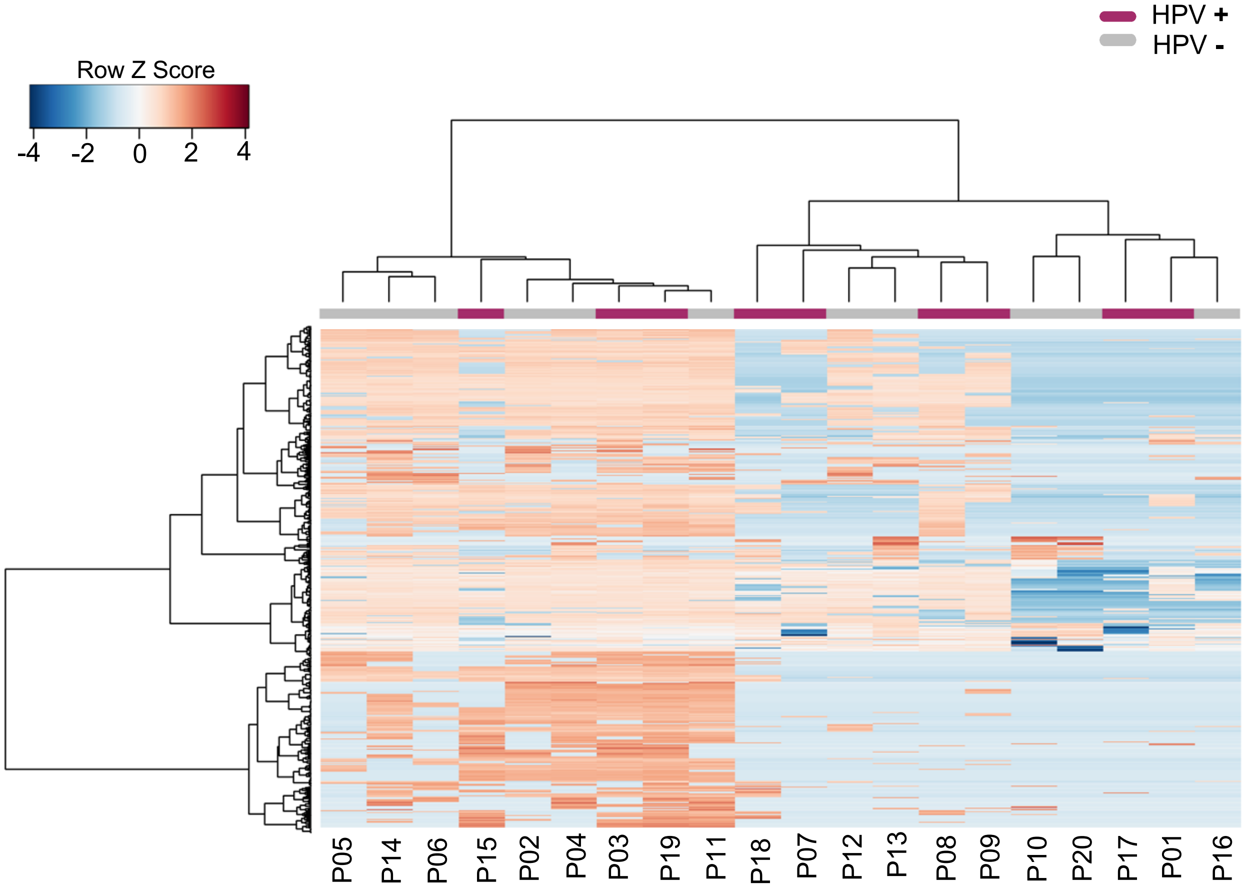

Supplementary Figure 3

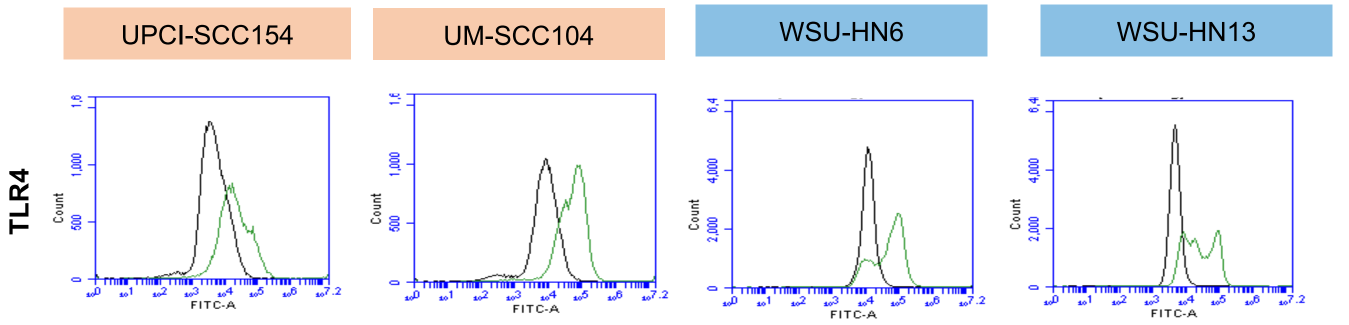

SUPPLEMENTARY TABLES

Supplementary Table 1. Sociodemographic and clinicopathological features of the patients included in the proteomics analysis.

Feature	***HPV(+)***	***HPV(-)***	***p value***
	n (%)		
**Age**			
Mean	34.7	32,7	
Median	37	35	*0.656*
Range	20-40	20-39	
**Sex**			
Male	6 (66,7)	6 (54,5)	*0.670*
Female	3 (33.3)	5 (45,5)	
**Tobacco consumption**			
Yes	5 (62,5)	10 (90,9)	*0.262*
No	3 (37,5)	1 (9,1)	
**Alcohol consumption**			
Yes	3 (37,5)	10 (90,9)	*0.041*
No	5 (62,5)	1 (9,1)	
**Anatomical site**			
Tongue	3 (37,5)	4 (40)	*0.84*
Floor of the mouth	3 (37,5)	2 (20)	
Other	2 (25)	4 (40)	
**T classification**			
T1/T2	0 (0)	3 (27,3)	*0.218*
T3/T4	9 (100)	8 (72,7)	
**N classification**			
N0	5 (55,6)	3 (27,3)	*0.362*
N1-N3	4 (44,4)	8 (72,7)	
**Histological differentiation**			
I	5 (55,6)	6 (54,5)	*0.055*
II	4 (44,4)	3 (27,3)	
III	0 (0)	2 (18,2)	
**Surgical margins**			
Negative	6 (85,7)	7 (63,6)	*0.596*
Positive	1 (14,3)	4 (36,4)	
**Treatment**			
Surgery	3 (33,3)	4 (36,4)	*1*
Surgery +RT	5 (55,6)	5 (45,5)	
Surgery +RT + CTX	1 (11,1)	2 (18,2)	
**Recurrence**			
Yes	7 (77,8)	6 (60)	*0.628*
No	2 (22,2)	4 (40)	

Abbreviations: HR-HPV (High-risk Human Papillomavirus), RT (radiotherapy), CTX, (chemotherapy).

Supplementary Table 2. Differentially expressed proteins between HPV+ and HPV - OSCC samples after Gene Ontology enrichment analysis, and miRNA prediction using samples in TCGA.

Gene	Protein	*p* value	Log2 ratio	miRNA fold change
Up-regulated proteins				
RACK1	Receptor of activated protein C kinase 1	**0.044**	0.61	-
CAD	CAD protein	**0.019**	1.99	-
RPL14	60S ribosomal protein L14	**0.002**	1.12	1.72
RPL29	60S ribosomal protein L29	**0.048**	0.68	-
EIF4A2	Eukaryotic initiation factor 4A-II	**0.038**	0.94	1.90
HNRNPF	Heterogeneous nuclear ribonucleoprote	**0.021**	0.77	-
SF3B3	Splicing factor 3B subunit 3	**0.029**	1.52	-
VARS	Valine-tRNA ligase	**0.019**	1.66	-
DYNC1H1	Cytoplasmic dynein 1 heavy chai1	**0.001**	1.49	-1.42
LRPPRC	Leucine-rich PPR motif-containing pro	**0.042**	1.02	3.11
CKAP4	Cytoskeleton-associated protein 4	**0.038**	1.50	2.00
COPS3	COP9 signalosome complex subunit 3	**0.023**	1.60	2.40
S100A8	Protein S100-A8	**0.004**	1.80	1.97
Down-regulated proteins				
RPL23	60S ribosomal protein L23	**0.039**	-0.99	-
RPS11	40S ribosomal protein S11	**0.023**	-1.85	-
RPS24	40S ribosomal protein S24	**0.037**	-0.975	-
EIF4A3	Eukaryotic initiation factor 4A-III	**0.004**	-2.03	-
NDRG1	NDRG1	**0.001**	-2.24	5.86
EIF4G2	Eukaryotic translation initiation factor	**0.008**	-1.14	3.11
PLP2	Proteolipid protein 2	**0.012**	-1.77	1.47
SERPINA1	Alpha-1-antitrypsin	**0.009**	-4.20	1.74
A2M	Alpha-2-macroglobulin	**0.004**	-4.47	1.85
HP1BP3	Heterochromatin protein 1-binding prot	**0.042**	-1.04	2.58
SON	SON	**0.016**	-1.52	-
PPP1R13L	RelA-associated inhibitor	**0.015**	-1.67	-
YWHAH	14-3-3 protein eta	**0.017**	-1.73	-

Supplementary Table 3. Enriched biological processes p-value.

Differential expressed proteins		
**Term**	**p-value**	**Adjusted p-value**
Viral process	4.09	0.01
Cellular protein metabolic process	3.58	0.01
Viral gene expression	2.38	0.01
Viral transcription	2.71	0.01
Protein targeting to ER	0.01	0.01
SRP-dependent cotranslational protein targeting to membrane	8.41	0.01
Cotranslational protein targeting to membrane	1.04	0.01
Nuclear-transcribed mRNA catabolic process	4.22	0.01
Nuclear-transcribed mRNA catabolic process, nonsense-mediated decay	7.97	0.01

HPV (+) exclusive proteins		
**Term**	**P-value**	**Adjusted P-value**
Gene expression	3.27	0.01
Nuclear export	2.10	0.01
Neutrophil degranulation	1.22	0.01
Neutrophil activation involved in immune response	1.39	0.01
Neutrophil mediated immunity	1.58	0.01
mRNA transport	5.10	0.01
mRNA-containing ribonucleoprotein complex export from nucleus	3.50	0.01
mRNA export from nucleus	5.58	0.01
RNA export from nucleus	1.61	0.01
RNA processing	3.90	0.01

HPV(-) exclusive proteins		
**Term**	**P-value**	**Adjusted P-value**
Negative regulation of blood coagulation	1.74	8.88
Receptor-mediated endocytosis	1.15	6.53
Humoral immune response mediated by circulating immunoglobulin	4.66	2.97
Complement activation, classical pathway	3.78	2.75
Regulation of protein processing	8.60	7.32
Regulation of acute inflammatory response	2.48	2.53
Regulation of protein activation cascade	1.95	9.98
Regulation of humoral immune response	5.40	9.18
Regulation of immune effector process	6.57	8.39
Regulation of complement activation	2.40	6.14

Supplementary Table 4. Correlation between S100A8 expression and clinicopathological features.

Variable	***Category***	***S100A8***		
		Low	***High***	***p***
Sex	*Male*	75.8	76.9	*0.917*
	Female	24.2	23.1	
Tobacco use	*Yes*	83.9	90.5	*0.494*
	No	16.1	9.5	
Alcohol use	*Yes*	64.5	66.7	*0.873*
	No	35.5	33.3	
Anatomical site	*Tongue*	43.3	50	*0.816*
	Floor m.	33.3	25	
	Other	23.3	25	
Tumor	*T1-T2*	36.4	11.5	***0.030****
	T3-T4	63.6	88.5	
Node	*N0*	0	0	*ND*
	N1-N3	100	100	
Clinical stage	I-II	24.2	0	***0.007****
	III-IV	75.8	100	
Histological classification	*I*	60.6	72	*0.448*
	II	30.3	16	
	III	9.1	12	
Surgical margins	*Negative*	96.3	71.4	***0.015****
	Positive	3.7	28.6	
Recurrence	*Yes*	48.5	26.9	0.092
	No	51.5	73.1	

** Statistically significant difference.*
